# Case Report: Return to Sport Following the COVID-19 Lockdown and Its Impact on Injury Rates in the German Soccer League

**DOI:** 10.3389/fspor.2021.604226

**Published:** 2021-02-18

**Authors:** Dhruv R. Seshadri, Mitchell L. Thom, Ethan R. Harlow, Colin K. Drummond, James E. Voos

**Affiliations:** ^1^Department of Biomedical Engineering, Case Western Reserve University, Cleveland, OH, United States; ^2^Case Western Reserve University School of Medicine, Cleveland, OH, United States; ^3^Department of Orthopaedic Surgery, University Hospitals Cleveland Medical Center, Cleveland, OH, United States; ^4^Sports Medicine Institute, University Hospitals Cleveland Medical Center, Cleveland, OH, United States

**Keywords:** COVID-19, Bundesliga, injury risk, return to sport, workload optimization, reduce injury burden

## Abstract

The Bundesliga made headlines for becoming the first major sports league to return to sport worldwide following COVID-19 lockdown. To-date, there lacks retrospective studies on longitudinal injury rates to elucidate the effect isolation measures had on the health and safety of professional athletes. This study sought to compare injury rates experienced by Bundesliga athletes before and after the COVID-19 lockdown. Data was collected from public injury and player reports regarding the Bundesliga, with injury defined as trauma resulting in loss of game time. Descriptive statistics were used to present differences in injury incidence between all Bundesliga Match days pre- and post-lockdown. Between the league's resumption and completion on May 16 and June 27, 2020, injuries occurred in 21 forwards (FW), 11 central midfielders (CM), 12 wide midfielders (WM), 16 central defenders (CD), 6 fullbacks (FB), and 2 goalkeepers. Players had 1.13 (95% CI 0.78, 1.64) times the odds of being injured following the COVID-19 lockdown, with a 3.12 times higher rate of injury when controlling for games played compared to injury rates pre-lockdown (0.84 injuries per game vs. 0.27 injuries per game). The most frequent injury group was muscular injuries with 23 injuries total, with 17% of athletes experiencing injury during their first competitive match following lockdown. Injury rate increased over 3-fold following COVID-19 lockdown. Athletes did not experience an increased rate of injury with more cumulative competitive matches played. High injury incidence for players yet to complete their first competitive match may imply suboptimal sport readiness following home confinement.

## Introduction

The coronavirus disease 2019 (COVID-19) pandemic has spread globally, forcing governing bodies to close commercial activities to avoid social gatherings in early 2020 (Pillay et al., 2020; Sarto et al., 2020). Elite sport leagues and major international sporting events have been suspended or delayed as part of this response worldwide (Pillay et al., 2020). While COVID-19 itself poses many potential health deficits to the infected athlete returning to play (Phelan et al., 2020), we are only beginning to understand the physical, social, and psychological impact of training during the pandemic. Emerging calls to action by sports scientists are warning returning sports leagues of an additional reason to exercise extreme caution: an elevated injury risk for the isolated athlete (Casais-Martinez et al., 2020; Sarto et al., 2020).

In response to insufficient training stimulus, some experts suggest a drop in athlete work capacity will occur in proportion to the length of time in isolation. Athlete detraining, defined as a partial or complete loss of training-induced morphological and physiological adaptation, occurs in the short- and long-term. Short term detraining (<4 weeks) includes a rapid decline in maximal oxygen uptake (VO_2max_), maximal cardiac output, and reversal of training-induced changes in fluid-electrolyte regulating hormones (Mujika and Padilla, 2000a). While muscle strength may not be affected in the short-term, training-induced changes in skeletal muscle cross-sectional area also reversed rapidly, which may predispose athletes to increased injury risk (Mujika and Padilla, 2000a; Sarto et al., 2020). Symptoms of long-term detraining (>4 weeks) include marked decreases in VO_2max_ and endurance performance, lower lactate thresholds, and gradual reduction of muscle force production (Mujika and Padilla, 2000b). A recent study by Sarto et al. postulated that detraining experienced by the confined athlete will result in impaired athlete performance and higher injury risk without appropriate rehabilitation and reconditioning (Sarto et al., 2020).

The closest direct translation of these predictions was the 4.5-month 2011 NFL Lockout, where Myer et al. reported a spike of 12 Achilles tendon injuries during the NFL preseason alone, compared to a full season average of 8 Achilles injuries (Myer et al., 2011). Sports league lockouts resulting from collective bargaining agreement negotiations give us insight into an offseason without normal access to a team's facilities, healthcare and strength & conditioning professionals, and coaches, a scenario similar to the COVID-19 lockdown (Myer et al., 2011). Unique to the COVID-19 lockdown and the history of sports competition, however, is the reduced workload or absence of training likely experienced by many players due to stay at home orders implemented worldwide (Casais-Martinez et al., 2020).

**Figure 1:**
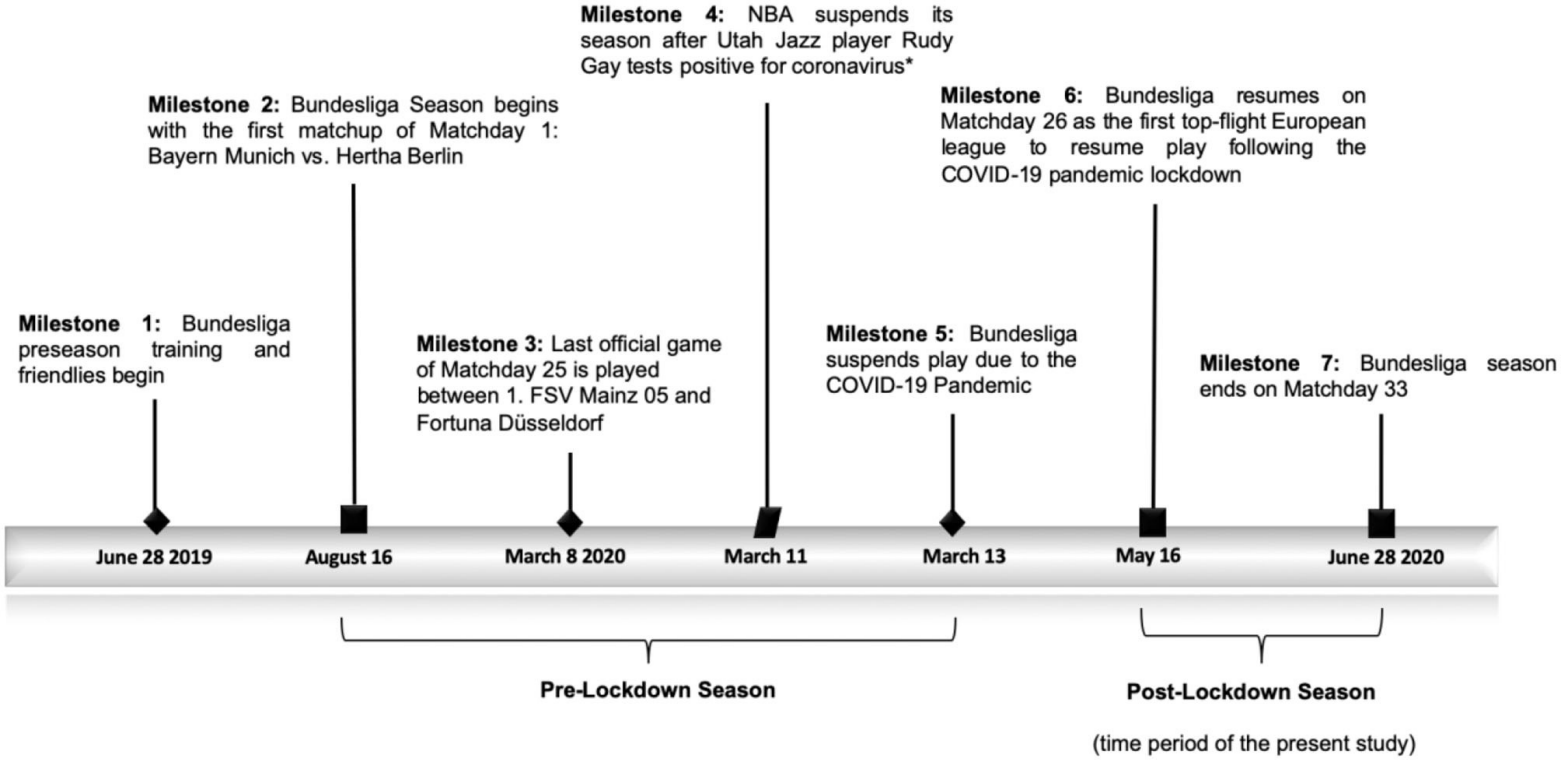
A timeline of the 2019–2020 Bundesliga Season surrounding the impact of the COVID-19 pandemic lockdown. *while not directly related to soccer, the NBA was the first major sports league to postpone their season due to the advent of the COVID-19 pandemic.

While the pandemic influenced lockdowns across Europe and the rest of the world (Oltermann, 2020), the elite German soccer league, *Bundesliga*, surprised sports fans and healthcare experts alike when it announced it would end its postponement, the first professional sporting organization to do so (Figure 1). The duration of the league lockdown (>4 weeks) would have resulted in both short- and long-term detraining of Bundesliga athletes, as the lockdown prevented access to organized team training activities (Mujika and Padilla, 2000a,b). Due to the increase in match frequency and hypothesized increase in internal and external player workloads (Seshadri et al., 2019a), the Bundesliga league expanded the number of in-game player substitutions (from 3 to 5 players) to mitigate the high risk of injury already present in the sport (Askling et al., 2013; Chena et al., 2019; Leventer et al., 2019). The 2019–2020 season injury rate in the Bundesliga prior to league suspension is known, with sports scientists describing the injury rate as 0.27 injuries per game (The Bundesliga Blueprint: early lessons from the return of German football TRACKADEMIC, 2020). Thus, the early return of the Bundesliga presents a challenge and a unique opportunity for sport scientists to better understand the impact of a long-term lockdown on athlete injury risk in a physically-demanding sport.

Therefore, the purpose of the present retrospective study was to compare injury rates of elite-level athletes before and after the Bundesliga COVID-19 lockdown. As a result of short- and long-term detraining, and more frequent competitive matches, the authors of this study hypothesized that the Bundesliga injury rate would increase following season suspension.

## Methods

### Participants

Five-hundred and thirty-seven players from the 2019–2020 Bundesliga season were represented in our dataset. Player game-loss injury reports were retrieved from publicly available data *transfermarkt* (https://www.transfermarkt.us/), a methodology previously used to quantify injury rates in Bundesliga players (Leventer et al., 2019). Player positions were categorized into forwards (FW), central midfielders (CM), wide midfielders (WM), central defenders (CD), fullbacks (FB), and goalkeepers. This study utilizes the definition of injury from the *transfermarkt* report [Bundesliga - Injured players (Detailed view), 2020], where injury was defined as any trauma or musculoskeletal injury causing loss of game time. This is notably a variation in the definition of injury as proposed by Fuller et al. (2006). Injury data was excluded for players whose date of injury was reported prior to May 16 – the date of season resumption – or following June 27 – the date of season completion. A detailed description of players injured and their respective injuries was recorded (Supplementary Table 1).

### Data Collection and Statistical Analysis

An odds ratio and 95% confidence internal was calculated for the likelihood of injury before and after the COVID-19 lockdown. Injury rate per game was inferred using the 82 remaining games of the season between Match days 26 and 34, with a total of 537 athletes studied. For these groups, a chi square analysis was performed to compare respective injury rates per game. For this analysis, the expected injury rate per game was set as 0.27, a number previously described for the initial 224 games of the season between Matchdays (e.g., a unit of time comprising all weekly matches) 1 and 25.

Using public gameplay reports for each injured player generated by the *SportsFan Ltd*-owned website *footballcritic* (FootballCritic - Football, 2020), the number of matches each player participated in prior to injury was recorded. A linear regression was performed representing the number of matches played prior to player injury. Chi square analysis was performed using the statistical programming language R. Figures were constructed using MATLAB_R2016b (The MathWorks, Inc, Massachusetts, USA).

## Results

The mean age of injured players was 26.8 ± 4.4 years with a mean body-mass-index (BMI) of 23.2 ± 1.2 (Height: 1.84 ± 0.06 m, Weight: 78.5 ± 6.9 kg). Seventy total game loss injuries were reported over the final 82 games played following league suspension, with 68 unique players reported injured (~12.6% of all athletes in the 2019–2020 Bundesliga league, compared to 11.2% of all athletes pre-lockdown). The odds ratio (95% Confidence Interval) suggesting the likelihood of a post-lockdown injury was 1.13 (0.78, 1.64). Injuries occurred in 21 forwards (FW), 11 central midfielders (CM), 12 wide midfielders (WM), 16 central defenders (CD), 6 fullbacks (FB), and 2 goalkeepers.

The injury rate per game following the COVID-19 lockdown was calculated to be 0.84 compared to 0.27 per game prior to the onset of COVID-19 (12.6% of 537 athletes in 82 games following the COVID-19 lockdown vs. 11.2% of athletes injured in 224 games). Athletes were 3.12 × more likely to have sustained injuries resulting in removal from play following the COVID-19 lockdown. Chi-square analysis demonstrated a significant difference between the injury rate post-lockdown with the injury rate pre-lockdown (χ^2^ = 164.84, p < 0.001).

**Figure 2:**
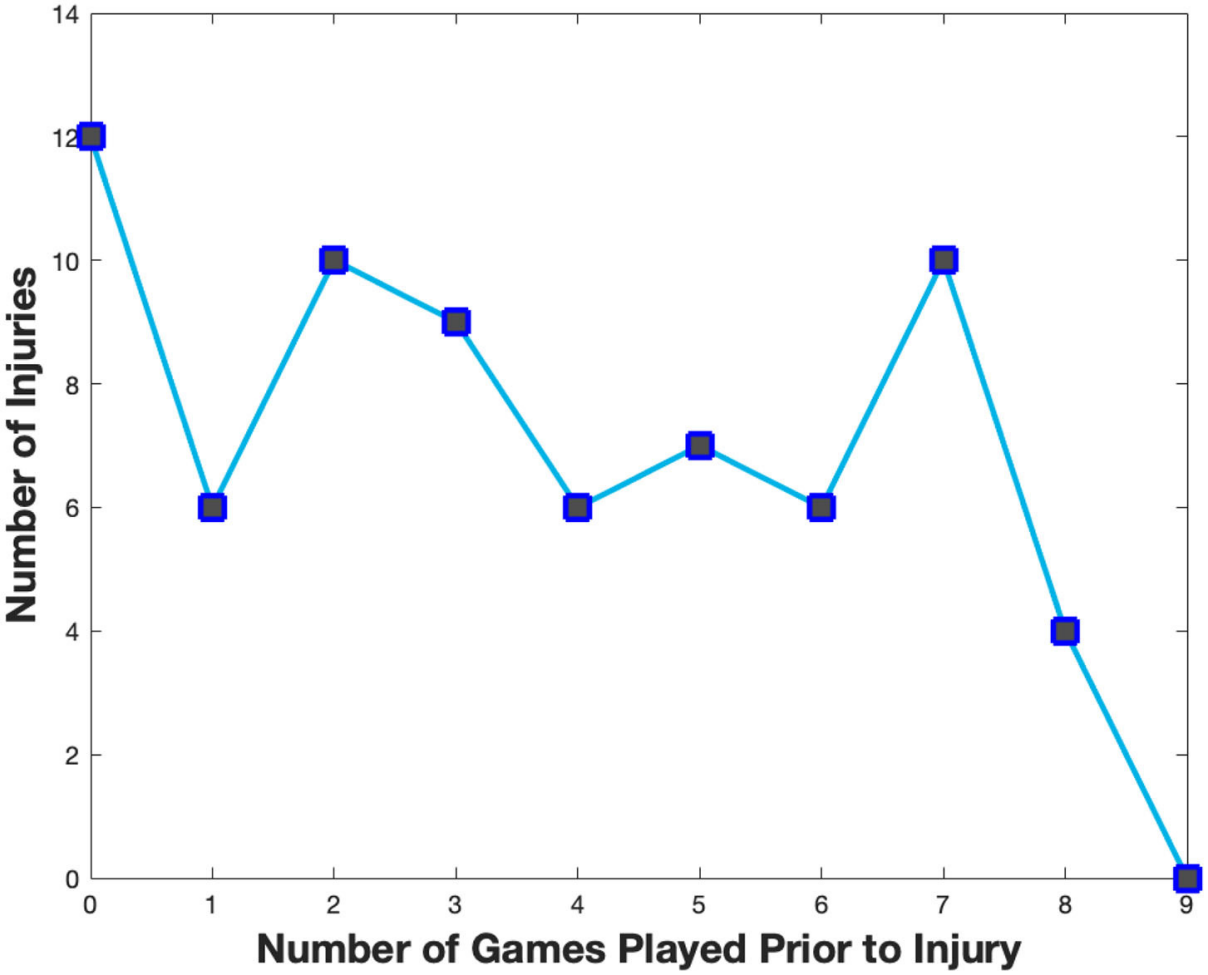
Comparative plot quantifying the number of matches played per each athlete before the onset of injury (r^2^ ~ 0.46 linear regression).

17.3% of all injuries occurred prior to competitive play initiation or during their first competitive match. The overall model fit for a linear regression between injury incidence and number of matches played prior to player injury was r^2^ = 0.42 (Figure 2). Muscular injuries (defined as: any report listing a muscle strain, tear, or problems associated with a muscle group or non-descript muscular injury) were the most common injuries noted among the Bundesliga athletes following the restart of the season (Table 1). Of these injuries, 57% were classified as non-descript in the affected muscle group; however, adductor or groin injuries were the next most common injury location at 21.7% of all muscular injuries.

**Table 1:** The relative percentage breakdown of injury group by position, where colors, ranging from green (low) to red (high), represent injury incidence per position (FW, Forwards; CM, Central Midfielder; WM, Wide Midfielder; CD, Central Defender; FB, Fullback).

	All^*^	**FW**	**CM**	**WM**	**CD**	**FB**
**Injury type**	**Percentage**					
Muscle	33%	24%	24%	33%	38%	50%
Knee	17%	14%	14%	33%	6%	17%
Ankle	13%	14%	9%	0%	25%	17%
Unknown	11%	19%	9%	8%	6%	17%
Fitness^**^	7%	14%	0%	0%	13%	0%
Hip	6%	0%	9%	8%	6%	0%
Foot	6%	0%	0%	8%	6%	0%
Back	4%	5%	0%	8%	0%	0%
Concussions	3%	10%	0%	0%	0%	0%

* *Includes the two injured goalkeepers, both with back injuries (n = 70 injuries in 68 athletes, ~12.6% of all athletes in the Bundesliga league)*.

** *Player or coaching decision to discontinue play following inadequate game aerobic fitness*.

## Discussion

This descriptive epidemiology study sought to assess whether the COVID-19 lockdown and subsequent stay-at-home orders initiated worldwide affected athlete performance in an adverse manner following the restart of the 2019–2020 Bundesliga season. At the time of writing, this retrospective study represents the first analysis informing orthopedic surgeons, team physicians, athletic trainers, and sports scientists of the impact the COVID-19 lockdown has had on the injury profile of elite-level athletes.

The increased rate of injuries is multifactorial in origin (Stern et al., 2020). One must consider, however, the influence of deconditioning from a prolonged competition holiday, training in a restrictive environment due to home confinement, an expedited preparatory phase, and a condensed competitive season on injury risk (Sarto et al., 2020). In order to improve, we must continue to find ways to measure data that meaningfully informs training staff on the physical condition of their athletes and their risk for injury (Seshadri et al., 2021). Interestingly, we found a proportionally high number of injuries in athletes either attempting to play in or train for their first match post-COVID. While it is not possible to draw a strict conclusion from this data, it may suggest a general miscalculation of athlete readiness for competition as a result of their RTP program (Gabbett, 2016).

While the overall injury rate both pre- and post-lockdown in the Bundesliga was relatively low, the data does provide professional and collegiate sports teams information to guide safe RTP protocols to mitigate injuries manifesting from over- or under-training. Changes in injury-risk have been previously studied among the Bundesliga league. Leventer et al. studied players with a first team contract in one of the 18 clubs in the first division of the Bundesliga league over a six-season period (3,438 injuries were documented; 40.6% match injuries and 59.4% training injuries) (Leventer et al., 2019). The IRR was significantly higher in the competitive season compared to pre-season across match (IRR: 2.00, 95% CI: 1.30–3.00) and training (IRR: 1.27, 95% CI: 1.11–1.43) injuries. Supporting and complementing the findings found in the literature (Leventer et al., 2019) and by our team suggest that further research is needed to confirm if players train in the preparatory phases in ways that might predispose them to an increase injury-risk as observed by the carry-over effect heading into matches.

Learnings from the 2011 NFL lockout suggested that there may have been an increase in relative reinjury risk during early sports reintegration, attributed to the greater residual biomechanical and neuromuscular deficits from deconditioning, prior injuries, or surgeries (Myer et al., 2011). Importantly, youth athletes many may go even longer periods of time (compared to the Bundesliga) before re-engaging in high-level sporting activity (Kelly et al., 2020). Specific athlete age, gender, and sport specializations have their own unique set of pre-dispositions to injury and they should be evaluated and rehabilitated appropriately prior to resuming competitive play (Post et al., 2019; Jayanthi et al., 2020). While the elimination of sports-related injuries is an unrealistic goal, the design of individualized conditioning protocols may help reduce the likelihood of sports-related injuries in all athletes. As the pandemic continues to have a global impact, it is important to pursue retrospective studies to provide team personnel with objective data on the biomechanical and physiological impacts of the COVID-19 pandemic on athlete performance and safety (Phelan et al., 2020; Sarto et al., 2020).

### Practical Application and Recommendations

**Figure 3:**
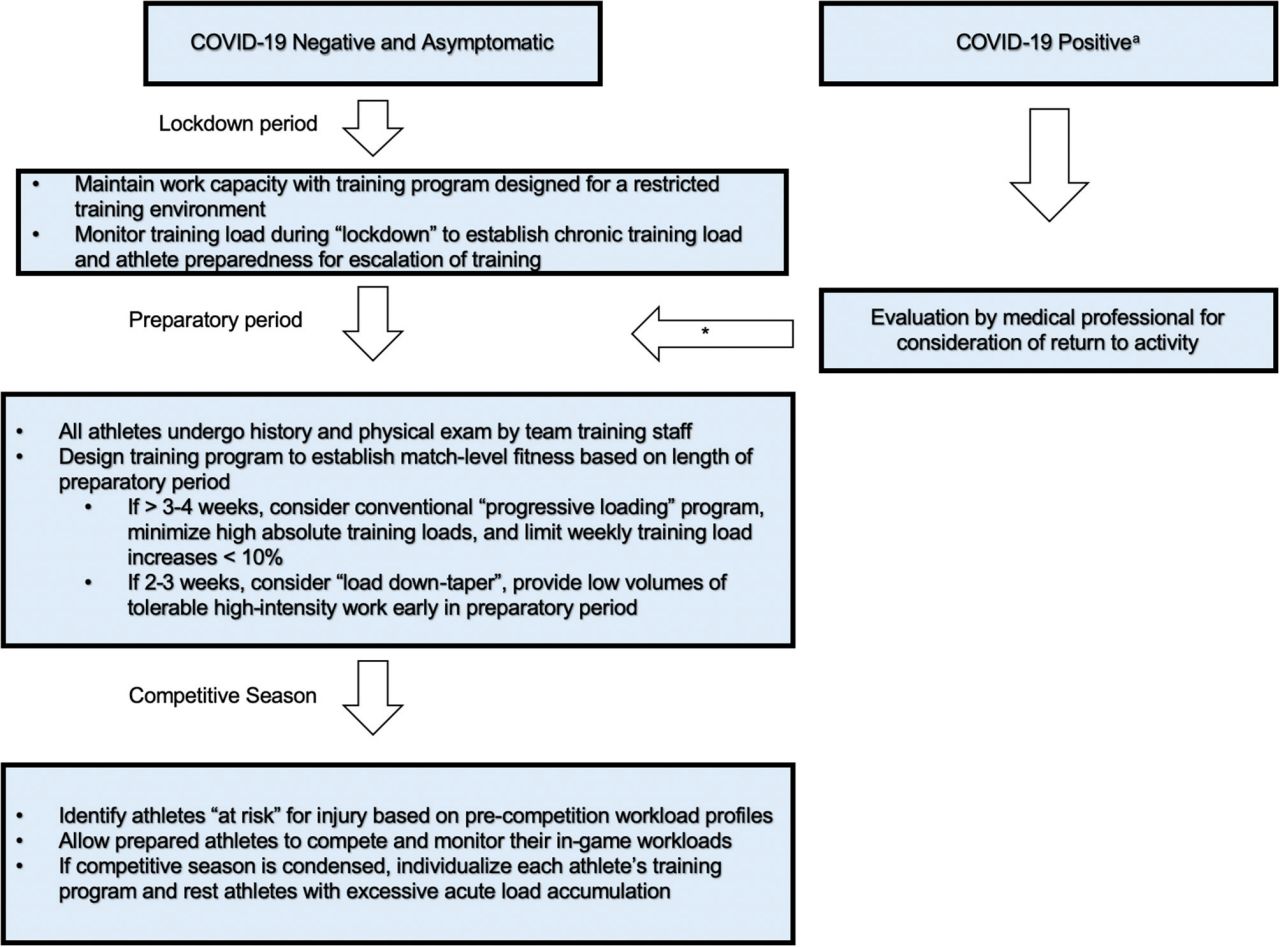
An example of a Return-to-Play strategy with focus on injury prevention following the COVID-19 lockdown. a: All athletes with positive testing should be isolated for 2 weeks regardless of symptoms. ^*^After appropriate treatment and medical clearance, athletes should slowly resume activity after 2 weeks of rest from symptom resolution under guidance of health care team. Figure adapted and modified from Seshadri et al. (2021).

Due to this increased injury rate following the COVID-19 lockdown, the authors of this study suggest an approach toward designing and monitoring RTP protocols for athletes following the lockdown and athletes who have tested positive for the virus using wearable sensor technology (Figure 3). Recent advancements in wearable sensor technology have utility in monitoring the internal, external, and physiological profile of athletes of all ages (Seshadri et al., 2021). In studying professional NFL athletes over a given season, Li et al. found that athletes who had an acute to chronic workload ratio (ACWR) higher than 1.6, as measured by tri-axial accelerometry data, were 1.5 times more likely to sustain an injury relative to time- and position-matched controls (64.6 vs. 43.1%; *P* = 0.004) (Li et al., 2017). We believe that the adoption of wearable sensor technology would enable a non-invasive, unobtrusive, and continuous modality for team physicians and athletic trainers to objectively assess the condition and health of professional, collegiate, and youth athletes as they RTP prior to the start of their respective season (Seshadri et al., 2019a,b; Seshadri et al., 2020). While further prospective studies are needed, wearable sensor technology and workload monitoring may play an effective role in injury prevention.

### Limitations of This Study

Our study is not without limitations. Data collection from public databases has inherent challenges with accuracy and specificity of reported data. As a result, our study may be inflating the incidence of certain injury types due to non-specific injury reporting. Additionally, without access to team data, the true number of injuries may be over- or under-estimated. When defining injury analogous to professional sporting organizations (Fuller et al., 2006) the injury rates derived from this descriptive epidemiology study are similar to those recently reported in unpublished studies. While the total number of injuries seen in the present study is relatively small relative to a typical Bundesliga season (Fünten et al., 2014), this report is the first of its kind, using the best available data to describe injury rate following the COVID-19 lockdown. Additionally, this study did not investigate, or correct for, the effect of increasing maximum in-game player substitutions.

## Conclusion

Bundesliga players were more likely to have sustained injuries following the COVID-19 lockdown, with many athletes experiencing injury during their return to competitive play. As professional sporting organizations continue to grapple with the repercussions of the pandemic, athletic trainers and coaching staff must be aware of a potential increase in risk for all injury types, with an emphasis on those of musculoskeletal origin. An increase in subjective and objective monitoring of the isolated athlete may mitigate the risk of injury related to both COVID-19 and its consequential physiological detraining during isolation.

## Data Availability Statement

Publicly available datasets were analyzed in this study. This data can be found here: https://www.transfermarkt.us/bundesliga/verletztespieler/wettbewerb/L1/plus/1; https://www.footballcritic.com/.

## Author Contributions

MT performed the data collection and analysis. DS, MT, and EH contributed to the writing of the manuscript, while CD and JV advised the project and contributed heavily to the editing of the manuscript. All authors contributed to the article and approved the submitted version.

## Conflict of Interest

JV serves as an educational consultant for Arthrex. The remaining authors declare that the research was conducted in the absence of any commercial or financial relationships that could be construed as a potential conflict of interest.
