# Supplementary material for: Case Report: Return to Sport Following the COVID-19 Lockdown and Its Impact on Injury Rates in the German Soccer League
Source: Front Sports Act Living. 2021 Feb 18;3:604226. doi: 10.3389/fspor.2021.604226 (PMC7931153; doi:10.3389/fspor.2021.604226)
Supplement: Supplementary file 1 [file Table_1.docx]

**Supplementary Table 1. List of athletes and recorded injury suffered during the 2019-2020 Bundesliga Season.** Forwards (FW), central midfielders (CM), wide midfielders (WM), central defenders (CD), fullbacks (FB), and goalkeepers.

| **Club** | **Position** | **Injury** |
| --- | --- | --- |
| Hertha BSC | CD | Tear in the abductor muscle |
| FC Schalke 04 | CM | MCL Injury |
| FC Schalke 04 | CD | Ankle Problems |
| FC Bayern Munchen | CD | Fitness |
| VFL Wolfsberg | FB | Ankle Injury |
| 1 FC Union Berlin | FB | Unknown Injury |
| 1 FC Koln | WM | Knee Problems |
| Hertha BSC | CD | Bruised Foot |
| Hertha BSC | WM | Strain |
| Hertha BSC | Goalkeeper | Back Trouble |
| SV Werder Bremen | CD | Hip problems |
| SC Freiburg | CM | Ankle Injury |
| Borussia Monchengladbach | FW | Bruised Ankle |
| Borussia Monchengladbach | CM | Torn Muscle Fiber |
| Rasenballsport Leipzig | FW | Ankle Injury |
| FC Schalke 04 | CM | Knee Injury |
| Borussia Dortmund | CM | Knee Injury |
| FC Bayern Munchen | CD | Ankle Injury |
| Rasenballsport Leipzig | CM | Adductor Problems |
| Hetha BSC | FW | Concussion |
| TSG 1899 Hoffenheim | CM | Adductor Problems |
| Borussia Monchengladbach | FW | Foot Injury |
| FC Augsburg | FB | Adductor Problems |
| Eintract Frankfurt | WM | Calf Injury |
| SV Werder Bremen | CM | Hip Injury |
| FC Augsburg | FB | Muscular problems |
| SC Freiburg | CD | Torn Muscle Fiber |
| FC Augsburg | FW | Muscular problems |
| SV Werder Bremen | CD | Fitness |
| 1 FC Koln | FW | Knee Problems |
| SV Werder Bremen | FW | Fitness |
| Bayer 04 Leverkusen | WM | Muscular problems |
| FC Schalke 04 | FW | Ruptured Knee Ligament |
| Hertha BSC | WM | Muscle Injury |
| SV Werder Bremen | WM | Bruised Hip |
| FC Schalke 04 | CD | Torn Muscle Fiber |
| SV Werder Bremen | FW | Fitness |
| FC Bayern Munchen | FW | Back Bruise |
| Bayer 04 Leverkusen | FW | Hamstring Injury |
| 1 FSV Mainz 05 | CD | Knee Injury |
| FC Bayern Munchen | CM | Groin Injury --> Surgery |
| Borussia Dortmund | CM | Muscle Injury |
| 1 FC Koln | FW | Thigh Problems |
| Eintract Frankfurt | FW | Fitness |
| Borussia Monchengladbach | FW | Tear in the abductor muscle |
| Borussia Monchengladbach | FW | Ankle Injury |
| 1 FSV Mainz 05 | FW | Concussion |
| Borussia Dortmund | CD | Muscular Problems |
| SC Paderborn 07 | CD | Torn Muscle Fiber |
| Bayer 04 Leverkusen | CD | Ankle Injury |
| Hertha BSC | WM | Bruised Foot |
| SV Werder Bremen | CD | Adductor Problems |
| FC Bayern Munchen | FW | Bruise |
| FC Bayern Munchen | CD | Ankle Problems |
| Borussia Dortmund | FB | Knee Problems |
| 1 FC Koln | WM | Coccyx Contusion |
| Borussia Dortmund | Goalkeeper | Back Trouble |
| 1 FC Koln | FW | Muscle Injury |
| Hertha BSC | FB | Torn Muscle Fiber |
| Rasenballsport Leipzig | WM | Knee Injury |
| SC Paderborn 07 | WM | Knee Injury |
| Eintract Frankfurt | WM | Knee Injury --> Surgery |
| Rasenballsport Leipzig | FW | Knee Problems |
| TSG 1899 Hoffenheim | WM | Unknown Injury |
| 1 FSV Mainz 05 | FW | Unknown Injury |
| 1 FSV Mainz 05 | FW | Unknown Injury |
| Borussia Monchengladbach | FW | Unknown Injury |
| Eintract Frankfurt | CM | Unknown Injury |
| Fortuna Dusseldorf | CD | Unknown Injury |
| TSG 1899 Hoffenheim | CD | Fractured Foot |
